# *Arabidopsis thaliana* Glyoxalase 2-1 Is Required during Abiotic Stress but Is Not Essential under Normal Plant Growth

**DOI:** 10.1371/journal.pone.0095971

**Published:** 2014-04-23

**Authors:** Sriram Devanathan, Alexander Erban, Rodolfo Perez-Torres, Joachim Kopka, Christopher A. Makaroff

**Affiliations:** 1 Department of Chemistry and Biochemistry, Miami University, Oxford, Ohio, United States of America; 2 Max Planck Institute of Molecular Plant Physiology, Potsdam-Golm, Germany; Purdue University, United States of America

## Abstract

The glyoxalase pathway, which consists of the two enzymes, GLYOXALASE 1 (GLX 1) (E.C.: 4.4.1.5) and 2 (E.C.3.1.2.6), has a vital role in chemical detoxification. In *Arabidopsis thaliana* there are at least four different isoforms of glyoxalase 2, two of which, GLX2-1 and GLX2-4 have not been characterized in detail. Here, the functional role of *Arabidopsis thaliana* GLX2-1 is investigated. G*lx2-1* loss-of-function mutants and plants that constitutively over-express GLX2-1 resemble wild-type plants under normal growth conditions. *Insilico* analysis of publicly available microarray datasets with ATTEDII, Mapman and Genevestigator indicate potential role(s) in stress response and acclimation. Results presented here demonstrate that *GLX2-1* gene expression is up-regulated in wild type *Arabidopsis thaliana* by salt and anoxia stress, and by excess L-Threonine. Additionally, a mutation in *GLX2-1* inhibits growth and survival during abiotic stresses. Metabolic profiling studies show alterations in the levels of sugars and amino acids during threonine stress in the plants. Elevated levels of polyamines, which are known stress markers, are also observed. Overall our results suggest that *Arabidopsis thaliana* GLX2-1 is not essential during normal plant life, but is required during specific stress conditions.

## Introduction

The glyoxalase pathway has been identified in a wide range of organisms including mammals [1], [2], plants [3], [4], yeast [5] and protozoa [6]. The pathway consists of two enzymes, Lactoylglutathione lyase (GLX1, 4.4.1.5) and Hydroxyacyl glutathione hydrolase (GLX2, 3.1.2.6) [7], [8]. This pathway is involved in chemical detoxification by converting acyclic alpha oxoaldehydes to their corresponding alpha hydroxyl acids [6], [9], [10]. Although various 2-oxoaldehydes can be used as substrates, methylglyoxal is thought to be the primary substrate [7]. Methylglyoxal (MG) is produced primarily by the enzymatic and non-enzymatic elimination of phosphate from glycolytic intermediates, including dihydroxy acetone phosphate and glyceraldehyde-3- phosphate [11], [12], [13], [14]. Other sources of MG include carbohydrate metabolism and lipid, acetone and threonine catabolism [15], [16].

**Figure 1:**
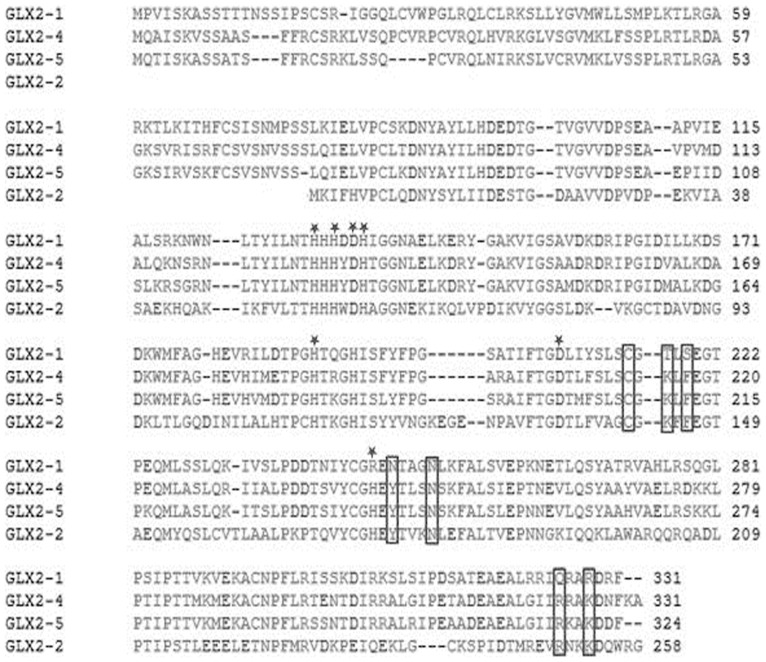
Sequence alignment of GLX 2-like enzymes of *Arabidopsis thaliana*. Metal binding amino acids are marked with *. Residues boxed represent substrate (SLG) binding amino acids.

GLX1 catalyzes the formation of S-D lactoyl glutathione (SLG) from methylglyoxal [17], [18], and GLX2 catalyzes the hydrolysis of SLG to form lactic acid and to regenerate reduced glutathione [19], [20]. GLX2 belongs to the β-lactamase family of proteins, which are metallo-enzymes that contain a number of highly conserved metal- and substrate-binding ligands [21], [22]. In humans a single gene encodes both cytoplasmic and mitochondrial forms of GLX2 [19]. In contrast, *Arabidopsis thaliana* contains four genes for putative glyoxalase II isozymes, three of which (*GLX2-1, 2-4, 2-5)* encode proteins that appear to be localized in the mitochondrion and one (*GLX 2-2*) that encodes a cytosolic protein [3]. A fifth GLX2- like enzyme, ETHE1, was recently shown to not have GLX2 activity, but acts as a sulfur dioxygenase [23], [24]. GLX2-2 and GLX2-5 have been extensively studied at the biochemical and structural levels [3], [25]. GLX2-2 binds zinc, iron and manganese while GLX 2-5 contains a Fe (III) Zn (II) center [3], [22], [25], [26], [27]. GLX2-1 exhibits high sequence similarity to GLX2-5, being 80% identical and 88% similar at the amino acid level (Figure 1). However, several highly conserved GLX2-2 and GLX2-5 metal and substrate-binding ligands are not conserved in GLX2-1. Specifically, GLX2-1 contains an arginine at the metal binding position 253 instead of a histidine (Figure 1). Surprisingly, GLX2-1 was found to still bind two equivalents of iron or zinc in its most stable form [28]. GLX2-1 also contains the amino acids R, N, Q, R, in place of the conserved SLG-binding residues H, Y, R, K at positions 246, 248, 325 and 328, respectively. Consistent with this observation, it was recently shown that unlike GLX2-2 and GLX2-5, GLX2-1 does not utilize SLG or several other glutathione analogues as substrates, but rather exhibits low-level β-lactamase activity [29]. Furthermore, replacement of R, N, Q, and R in GLX 2-1 with H, Y, R, and K at positions 246, 248, 325 and 328, respectively, generates catalytic activity for SLG [30]. Therefore, even though GLX2-1 exhibits high sequence similarity with GLX2 enzymes, it is not a GLX2.

To gain insights into the functional role of Arabidopsis *GLX2-1,* plants in which *GLX2-1* was either inactivated by a T-DNA insertion (*glx2-1*), or in which *GLX2-1* is constitutively over-expressed (GLX2-1OE) were studied. The plants were examined under normal conditions and under several different abiotic stress conditions, and in the presence of excess exogenous amino acids. Finally, non-targeted metabolic profiling was performed to determine if variations in *GLX2-1* expression can induce a hidden metabolic phenotype, and if so, what metabolites change when *GLX2-1* expression is altered. In this report we show that *Arabidopsis thaliana GLX2-1* is a non-essential gene, but that it plays an important role in stress response. *GLX2-1* expression is elevated during abiotic stress conditions, and *glx2-1* plants are sensitive to abiotic stress conditions. Also, variations in *GLX2-1* expression affects the levels of several amino acids and other primary metabolic intermediates in *Arabidopsis thaliana,* which could directly impact its ability to respond to stress.

## Materials and Methods

### *In silico* analysis of *GLX2-1* Expression

To understand the expression profile of *GLX2-1,* we performed an expression analysis using data from multiple public databases using various *insilico* tools to understand the role(s) of *GLX2-1*. Analysis of expression patterns of GLX2-1, as well as *GLX2-2* and *GLX2-5*, two bonafide GLX2 enzymes was performed using Genevestigator [31]. Analysis of co-expressed gene networks was performed using ATTED II [32]. Finally, to understand the potential metabolic impact of alterations in *GLX2-1* expression, we used MapMan, which is a tool that can display large datasets such as gene expression data onto diagrams of metabolic pathways or other processes [33]. In this study, we conducted a meta-analysis of manually curated microarray data available from public databases.

### Plant lines and Growth Conditions

*Arabidopsis thaliana* Wassilewskja (Ws) and Colombia (Col) plants were used in this study. For growth on plates, seeds were surface sterilized with 70% ethanol followed by 15% bleach treatment for 20 min. After 5 rinses in water they were plated on half-strength MS medium plates that were then incubated at 4°C for 2 days for vernalization. The seeded plates were then transferred to a growth chamber (23°C, 16 h day/8 h night cycles) in the vertical position for root and shoot analysis, and the horizontal position for other studies. For studies involving plant growth in soil, the seeds were surface sterilized and sown on metro mix. Plants that contain a T-DNA insertion in *GLX2-1* (SALK_059108.49.95.X) in the Colombia background were obtained from the Arabidopsis Resource Center.

The effect of anoxia was tested by placing MS plates containing 8-day-old seedlings in an airtight canister in which an oxygen abstraction pack (ANAEROGEN, Oxoid, UK) was placed. Oxygen concentration and temperature were monitored using an indicator from ANAEROGEN and thermometer, respectively. The Anaerogen pack reduced the oxygen concentration to <1% in 30 min [34]. The canister was stored at 23°C in the dark for 24 h after which time the plates were exposed to the atmosphere in a sterile hood for 1 h. The plates were then closed and placed in a growth chamber for either for 12 h or 24 h, after which observations were made. Osmotic and salt stress was induced by growing plants in MS media containing 50, 100 and 200 mM mannitol or NaCl, respectively. The effect of cold and heat stress was tested by growing plants for eight days on half strength MS plates under normal conditions and then moving them to 4°C or 28°C for 48 h, respectively.

The effect of dark stress was tested by growing plants for eight days in half strength MS media under normal conditions and then wrapping them in aluminum foil for 48 h. The effect of stress by individual amino acids was tested by growing plants on plates containing MS media to which filter-sterilized amino acids were added prior to pouring the plate. The following amino acid concentrations were tested: L-threonine, L-serine: 0.1, 0.5, and 1.0, 2.0 mM, L-methionine: 0.2, 0.3, 0.4 mM, L-aspartate, L-lysine: 1.0, 1.5 mM, D-threonine, D-serine and D-aspartate: 2 mM.

### Molecular Studies

Plants homozygous for a T-DNA insertion in *glx2-1* were identified by PCR genotyping using a left border primer (ATGGTTCACGTAGTGGGCCATC) and a gene specific primer (GS1: ATTGAGCAGCAAGAATTGGA) or two gene specific primers (GS1 and GS2: AGGGTACCACAGGATAAGC). Over-expression of *GLX2-1* was achieved by cloning a PCR amplified *GLX2-1* cDNA fragment into pFGC 5941 (http://www.chromdb.org), which contains the modified 35S promoter from cauliflower mosaic virus. After sequence verifying for any mutations, the construct was introduced into *Agrobacterium tumefaciens* GV3101 by heat shock. Arabidopsis plants of the Ws ecotype were transformed [35]. Transgenic seedlings were selected by their resistance to BASTA (Glufosinate ammonium) and confirmed by PCR. Total RNA was isolated using a Qiagen RNEasy Kit, and *GLX2-1* transcript levels were determined by qRT- PCR using 1.5 µg of total RNA. Thermal cycler settings for RT-PCR were as follows: 10 min at 65°C, 1 h at 50°C, 5 min at 85°C. Real-Time PCR assays were carried out using a qScript One-Step SYBR Green qRT-PCR Kit for iQ from Quanta Bioscience using a Bio-Rad iCycler. The settings for real time one step RT-PCR were as follows: 95°C for 30 s followed by a melt curve analysis consisting of 40 cycles of 3 s at 95°C, 30 s at 60°C, increasing 0.5°C per cycle after cycle 2. Experiments were conducted in triplicate on three independent pools of seedlings for the various conditions tested and standardized to β-tubulin.

### Plant Metabolite Profiling

Eight-day-old plate grown seedlings (+/− 2 mM L-Threonine) were harvested and 60 mg fresh weight (+/− 5%) was snap frozen in liquid nitrogen. Six replicates were prepared for each condition. The samples were homogenized using a bullet blender (Next Advance, USA) while frozen. Samples were prepared and analyzed essentially as described [36], [37]. Specifically, 360 µL of a precooled premix (300 µL of GC grade methanol, 30 µl of 2 mg/mL nonadecanoic acid methyl ester in chloroform, 30 µl of 0.2 mg/mL ribitol in methanol) was added to the samples. After 15 min of shaking at 70°C, 200 µl of chloroform was added and the samples incubated at 37°C for 5 min. 400 µl of water was added to this mixture, then vortexed and centrifuged at 14000 rpm for 5 min. 160 µl from the upper polar phase was carefully withdrawn and transferred to a new 1.5 ml micro centrifuge tube and dried using speed vacuum without heating. Samples were stored at −20°C until use. Metabolite samples were chemically derivatized by methoxyamination and trimethylsilylation; samples were incubated with 40 µL of 40 mg methoxyaminehydrochloride per mL pyridine for 1.5 h at 37°C, and afterwards 80 µL of BSTFA-Premix (including 7/1 BSTFA and Alkane-Mixture) [36] was added. Measurements were done with an Agilent 6890N24 gas chromatograph (Agilent Technologies, Böblingen, Germany; http://www.agilent.com) with splitless injection onto a FactorFour VF-5 ms capillary column of 30-m length, 0.25-mm inner diameter, 0.25-µm film thickness (Varian-Agilent Technologies) plus 10 m integra guard, which was connected to a Pegasus III time-of-flight mass spectrometer (LECO Instrumente GmbH, Mönchengladbach, Germany; http://www.leco.de). All analyses were conducted in splitless mode with 1 µL injection volume with helium as carrier gas at a flow rate of 0.6 mL/min. Injector temperature at 230°C, initial 70°C for 1 min and then ramped to 350°C at the rate of 9°C/min and held at 350°C for 5 min.

### Metabolite identification, quantification and statistical analysis

Relative quantification and identification of metabolites were carried out using Tagfinder software [38], [39]. Compound responses, i.e. baseline corrected peak heights, were volume corrected for errors during sample preparation or GC injection using the ribitol standard and normalized by the fresh weight of each sample. First, relative responses of each metabolite were calculated relative to the mean of all samples per analyte, and subsequently log _(10)_ transformed. Statistical significance was assessed by applying student’s t-test at P ≤ 0.05 significance to the log _(10)_ transformed ratios. Independent component analysis was carried out using the web tool at http://metagenealyse.mpimp-golm.mpg.de/ [40] for initial visual analysis of the main variances in the metabolite fingerprinting data set. All other statistical analyses were performed using Multiple Experiment Viewer of the TM4 suite [41]. For plant shoot length, germination and qPCR, students- t-test was performed. P<0.05 was considered significant for all.

## Results

### Glyoxalase 2-1 is not essential for normal growth and development

The role of GLX2-1 in plant growth and development was evaluated by examining the effect of inactivation and over-expression of *GLX2-1*. A T-DNA insertion line (SALK_059108.49.95.X) containing an insert in exon 4 of *GLX2-1* was obtained from the ABRC (Columbus, OH). *GLX2-1* transcripts were absent from plants homozygous for the insert indicating the complete disruption of *GLX2-1* (Figure S1). Inactivation of *GLX2-1* did not have a noticeable effect on plant growth and development as *glx2-1* plants were indistinguishable from wild type plants under normal growth conditions (data not shown).

The effect of over-expression of *GLX2-1* was examined by generating transgenic plants that express *GLX2-1* from the 35S promoter. T2 generation plants from six independent transgenic lines were analyzed for *GLX2-1* transcript levels using RT-PCR and for possible morphological/phenotypic alterations. Similar to the results with *glx2-1* plants, no differences were observed between wild type plants and those containing elevated *GLX2-1* transcript levels (data not shown).

### *GLX2-1* is induced in response to abiotic stress

In the absence of any visible phenotype in over-expressing and knockout plants, an analysis of available *GLX2-1* expression data was performed using various *insilico* tools as a logical next step towards understanding the role(s) of *GLX2-1*. Analysis of expression patterns using Genevestigator [31] showed that *GLX2-1* transcript levels are elevated during several abiotic stresses, including: anoxia, hypoxia, drought, light, osmotic and salt (Figure S2). Furthermore, comparison of *GLX2-1* expression profiles relative to *GLX2-2* and *GLX2-5*, two bonafide GLX2 enzymes, showed that the three genes exhibited relatively similar expression patterns; in particular they are all induced by abiotic stresses (Figure S3). Furthermore, analysis of co-expressed gene networks using ATTED II [32] also showed that *GLX2-1* is generally co-expressed with stress response genes (Figure S4). Consistent with these observations, a number of potential cis-regulatory elements associated with stress inducible genes were found upstream of *GLX2-1* (Figure S5), including the Gibberellin Responsive Element (GARE), ANAERO3, a consensus motif found in the promoters of anaerobic genes involved in the fermentative pathway, ARF, Auxin Response Factor [42], and ERELEE4 ERE, an Ethylene Responsive Element [43]. The involvement of these motifs in stress-induced transcription of a number of genes has been well documented [44], [45], [46], [47], [48].

Finally, analysis of *GLX2-1* using MAPMAN [33], a pathway analysis tool designed to visualize and interpret microarray datasets, suggested that *GLX2-1, GLX2-2* and *GLX2-5* might have a role in the degradation of amino acids, specifically those belonging to the aspartate family (Figure S6). Taken together, these results indicate that even though GLX2-1 does not hydrolyze SLG, and therefore is not a GLX2 enzyme, it is co-expressed with GLX2 enzymes and may play a role in stress response and/or in the detoxification of byproducts from amino acid degradation.

**Figure 2:**
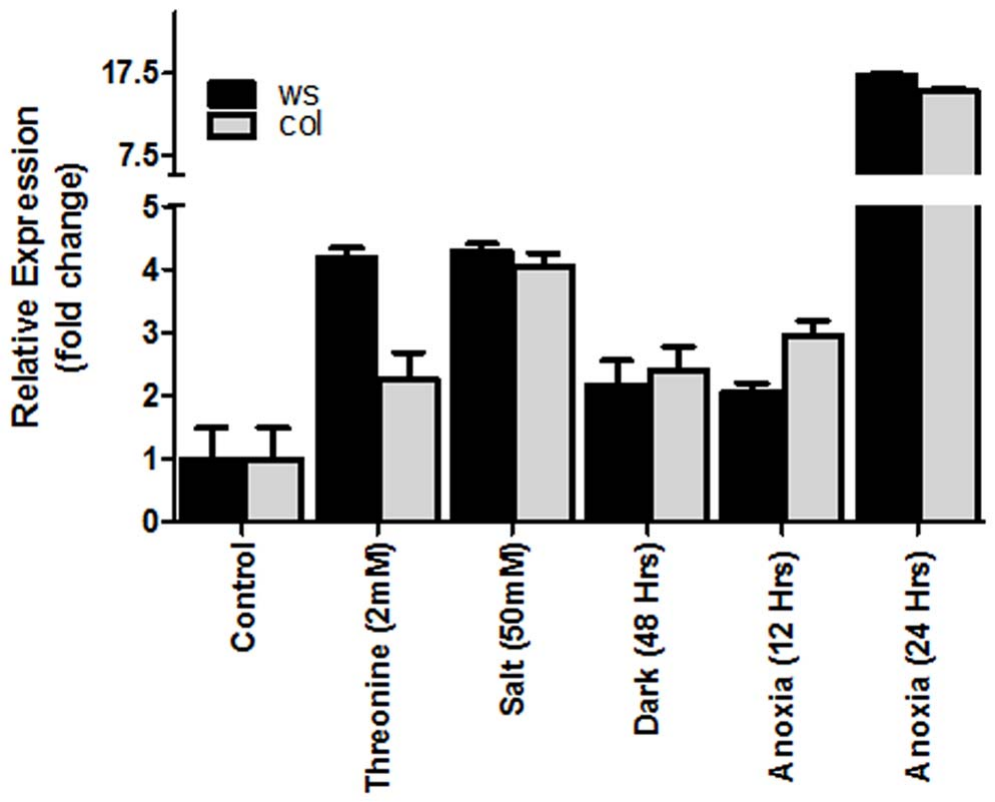
Relative expression levels of *GLX2-1* in response to various stresses. RNA was harvested from plants subjected to various stresses: Threonine or NaCl for 8 days, dark for 48 hours, anoxia for 12 or 24 hours followed by 24 hour recovery. Control plants were grown for 8 days under normal light and temperature conditions. Data are mean ± S.D (in triplicate) of expression analysis under each condition.

Since the increase in *GLX2-1* transcript levels under the various stress conditions was relatively modest (log_2_<2) in the microarray experiments, qRT-PCR was performed on wild type plants of two different ecotypes, *Col* and *WS*, that had been subjected to different stress conditions, to confirm the results. *GLX2-1* transcript levels were increased when plants were exposed to stress conditions, with C*olumbia* and *WS* plants exhibiting generally similar responses, which are consistent with expression profiling data, (Figure 2). Exposure of plants to darkness for 48 hours resulted in an approximate two-fold increase in *GLX2-1* transcript levels, while *GLX2-1* transcript levels increased approximately four-fold when plants were subjected to salt stress (Figure 2). Exposure of plants to anoxic stress for twelve hours had no effect on *GLX2-1* levels (data not shown), however, *GLX2-1* transcript levels increased approximately two-fold after a 24 hour recovery period following anoxic stress. Exposure of the plants to anoxic conditions for 24 hours followed by a 24 hour recovery period resulted in an approximately 15 fold increase in *GLX2-1* transcript levels (Figure 2). This result confirms that *GLX2-1* is induced during abiotic stress and suggests that it may have a role in stress recovery.

### *GLX2-1* is required for stress-response

**Figure 3:**
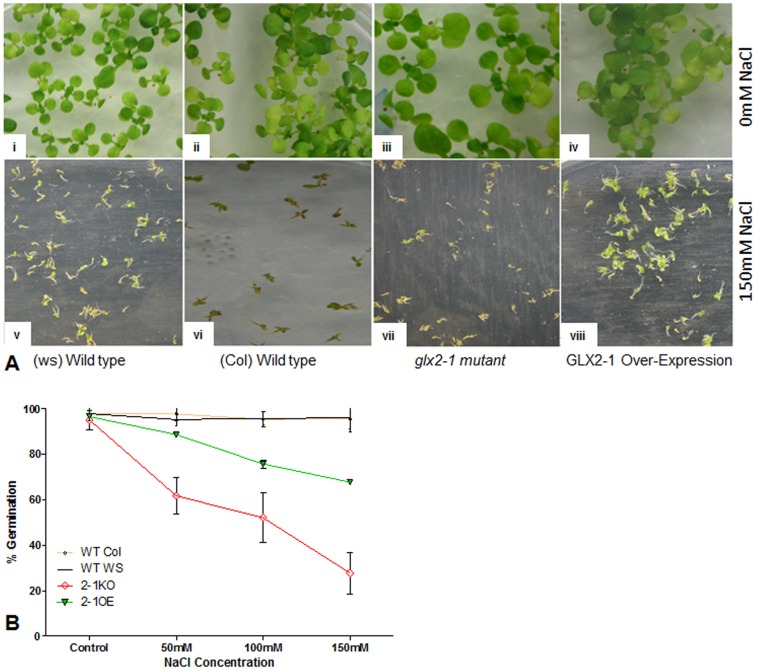
Arabidopsis *glx2-1* plants are sensitive to salt stress. (A) Representative images of seedlings grown in the absence (top panels) and presence (bottom panels) of 150 mM NaCl. (B) Graphical representation of germination frequency (ungerminated vs germinated seeds). Results from 4 independent trials containing ∼400 seeds in each trial are shown. Data are mean ± S.D percent number of seeds germinated under each condition.

Our observation that *GLX2-1* transcripts are elevated under certain stress conditions suggested that over-expression of *GLX2-1* may alter the ability of plants to withstand certain stresses. Therefore, the effect of exposure of wild type, *glx2-1* and *GLX2-1* OE plants to several stresses, including threonine, salt, dark, anoxia, osmotic, heat, cold, and salt was tested. In agreement with previous studies, wild-type plants subjected to osmotic, heat, cold, and salt stress exhibited reduced germination rates, stunted growth, and increased lateral formation roots [49]. The response of GLX2-1OE and *glx2-1* plants to osmotic, heat and cold stress was similar to that observed for wild type plants (data not shown). However, *glx2-1* plants are more sensitive to salt stress than wild type plants (Figure 3 A, B). The presence of 150 mM NaCl in the germination medium had only a minor effect on the germination of wild type seeds with a 20% decline in germination frequency being observed. In contrast, the germination of *glx2-1* seeds was reduced at all salt levels and was approximately 40% of control levels on 150 mM NaCl plates. The presence of salt had little to no effect on the germination rate of *GLX2-1*OE seeds (Figure 3 A, B).

**Figure 4:**
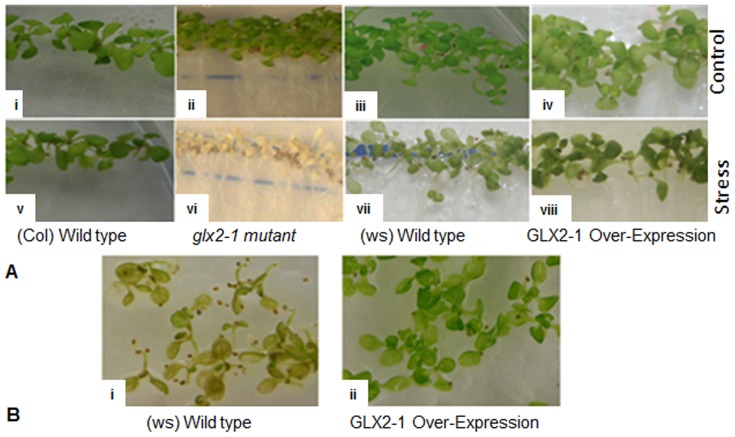
Arabidopsis *glx2-1* plants are sensitive to anoxia. Eight-day-old seedlings were exposed to low oxygen conditions for 24 hours in the dark. Images were taken after a 24 hr. recovery period in the growth chamber (A). Wild type and GLX 2-1OE plants after 72 hours of recovery, following 24 hours anoxia (B). Representative image from multiple independent trials is shown.

Differences were also observed in the response to anoxic stress. Wild type (Col) and *GLX2-1*OE plants exhibited no adverse effects when exposed to anoxic conditions for 24 h, either immediately after exposure (data not shown), or after a 24 h recovery period (Figure 4A-v, viii). In contrast, the same conditions were lethal to *glx2-1* plants (Figure 4A-vi). Although *glx2-1* plants looked normal and healthy immediately after the 24 h anoxia treatment, chlorosis and plant death occurred during the 24 h recovery phase. Furthermore, wild type plants were less tolerant to anoxic stress than *GLX2-1OE* plants. Wild type plants exhibited considerable cell death and chlorosis after recovering for 72 h after a 24 h exposure to anoxic conditions. In contrast GLX2-1OE plants appeared green and healthy (Figure 4B-i, ii)

**Figure 5:**
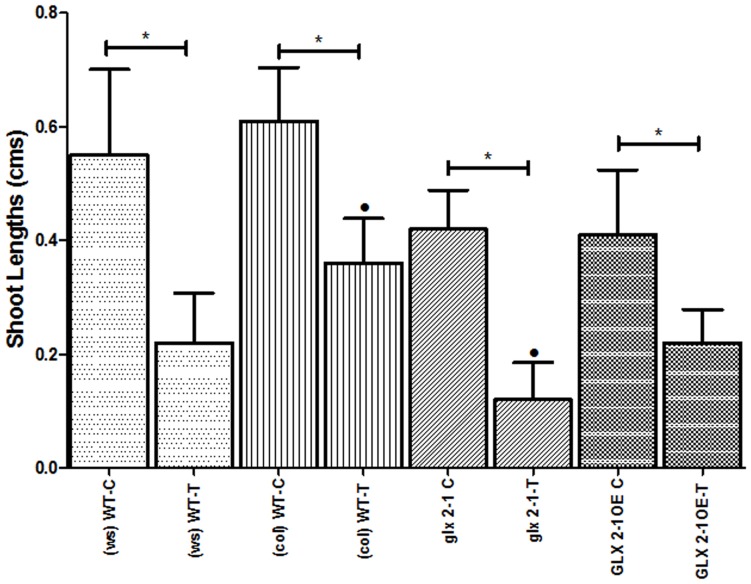
The effect of 2- threonine on shoot length. Shoot lengths of seedlings grown in the presence or absence of 2∼ 30 seeds in each trial are shown WT- Wild type, *glx2-1* – mutant plant, GLX2-1OE – Plants constitutively over-expressing the *GLX2-1* gene; C- Control, S-Stress; Ws-Wassilewskija ecotype and Col-Colombia ecotype. *indicates P < 0.05, students't-test.

The MAPMAN analysis predicted that *GLX2-1* may play a role in amino acid degradation. To test this hypothesis, the growth properties of wild type, *glx2-1* and *GLX2-1*OE plants in the presence of varying concentrations of several different amino acids, including L-threonine, L-serine, L-methionine, L-aspartate, L-lysine, D-threonine, D-serine and D-aspartate were compared. The presence of exogenous amino acids in the growth media had an effect on the timing and rate of seed germination, as well as shoot and root growth of all the plants. In most instances no differences in growth were observed among wild type, *glx2-1*, *GLX2-1*OE seeds (data not shown); however, *glx2-1* plants were more sensitive to the presence of 2 mM L-threonine. In particular, the shoot lengths of *glx2-1* plants were reduced relative to wild type and *GLX2-1OE* plants when grown in the presence of 2 mM threonine. The shoots of *GLX2-1OE* plants were marginally shorter than wild type plants under control conditions and were reduced by exogenous threonine. However, the relative reduction in shoot length was less in *GLX2-1OE* plants than in either wild type or *glx2-1* plants (Figure 5). Consistent with these observations, a four-fold increase in *GLX2-1* transcript levels was observed in wild type plants grown in the presence of 2 mM threonine (Figure 2).

### Changes in *GLX2-1* levels and threonine stress result in alterations in metabolism

Our results show that GLX2-1 plays a role in plant stress response and suggest a possible role in threonine metabolism. However, the specific biochemical role of *GLX2-1* is not clear. It is also unclear why or how excess threonine inhibits shoot growth. Therefore, to gain further insights into the metabolic role of GLX2-1 and the effect of excess threonine on plant growth, metabolite profiles of wild type, *GLX2-1*OE and *glx2-1* plants grown either in the absence or presence of 2 mM L-threonine were compared. An established gas chromatography–mass spectrometry (GC-MS) protocol [37], [50] was used that enabled the qualitative and quantitative identification over 80 metabolites, plus an additional 40 repeatedly occurring, yet unidentified, mass spectral tags. Since the specific interest was in metabolites associated with threonine metabolism, the analysis focused on the polar metabolite fraction, which was enriched for small primary and secondary metabolites.

**Figure 6:**
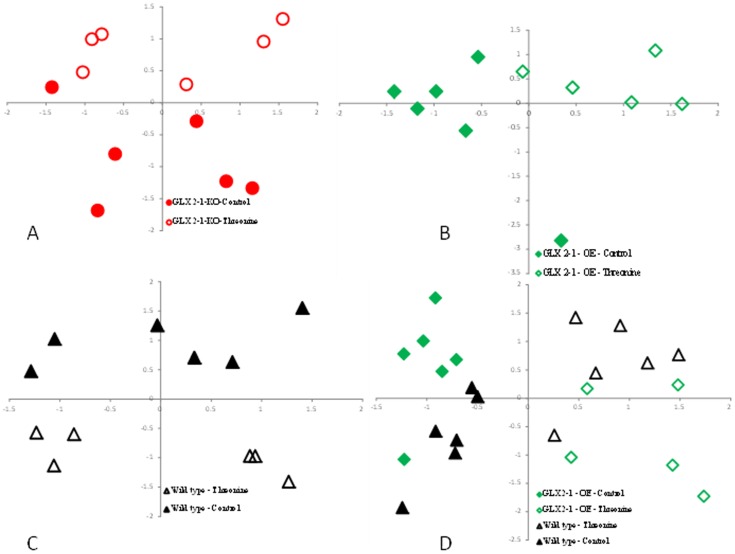
Independent component analyses of polar metabolite fingerprints from *A.thaliana glx2-1,* GLX2-1-OE, and wild type plants. Comparison between *glx2-1+/−* Threonine (a), GLX2-1-OE +/− Threonine (b), Wild type +/− Threonine (c) and GLX2-1OE and Wild type +/− threonine (d) are shown. Independent component analyses scores (IC 01 on X-axis and IC 03 on Y-axis) demonstrates common differences in seedlings in response to *GLX2-1* levels and/or exogenous Threonine stress.

The primary metabolite profiles obtained from GC-MS analysis revealed that subjecting the plants to exogenous threonine leads to significant changes in both primary and secondary metabolites, including organic acids, sugars, amino acids, and polyols. Independent component analyses (ICA) of the metabolite profiles of eight-day-old seedlings clearly discriminates among the plants tested under the different growth conditions (Fig. 6). A pair-wise comparison between threonine stressed and unstressed plants (wild-type, *glx2-1*, and GLX2-1 OE) was performed to directly compare the effect of threonine on metabolism (Figure 6 a, b and c). Comparisons of GLX2-1OE plants and wild type plants grown under normal as well as stress conditions were also performed (Figure 6 d). All of the analyses resulted in differential groupings based upon genetic background and treatment, but direct comparison between the *glx2-1* and wild type plants and *between the glx2-1* and *GLX2-1*OE plants was not performed at this time because the *glx2-1* mutation is in the Colombia ecotype and *GLX2-1OE* plants are in the Ws ecotype. Differences in ecotype has been shown to add to the variability of metabolic profiles [51]. Relative differences in metabolites and mass spectral tags expressed as fold changes are presented in Table 1 and Table S1, respectively, with statistically significantly (P≤0.05, student's t test) values bolded.

**Table 1:** Variations in metabolite levels between plants grown under different conditions and differential for GLX2-1 expression.

Metabolite	FC (WTT/WTC)	FC (OET/OEC)	FC (KOT/KOC)	FC (OEC/WTC)	FC (OET/WTT)
	*(n = 6)*	*(n = 6)*	*(n = 6)*	*(n = 6)*	*(n = 6)*
**Acids**					
Citric acid	−4.41	90.18	3.81	−68.82	−37.96
Fumaric acid	−35.09	146.86	−5.60	−72.15	5.90
Glutaric acid, 2-oxo-	179.74	291.57	107.69	−46.61	−25.26
Hexanoic acid, 2-ethyl-	−80.02	3.83	−53.57	−81.12	−1.88
Malic acid	−24.26	153.54	18.33	−78.17	−26.92
Malonic acid	−85.98	−19.17	153.20	−84.95	−13.23
Quinic acid	24.15	NA	4.30	NA	−69.40
**Alcohols**					
Hexadecan-1-ol, n-	5.98	−13.40	−14.24	17.58	−3.93
**Amino Acids**					
Alanine, 3-cyano-	13.04	82.92	49.52	−35.65	4.12
Alanine, beta-	−**38.62**	39.72	32.25	−**56.08**	−0.02
Aminomalonic acid	8.30	NA	−71.80	NA	−46.32
Asparagine	50.20	−34.80	162.91	4.58	−**54.61**
Aspartic acid	−27.72	−38.81	**35.60**	−**46.57**	−54.76
Butanoic acid, 4-amino-	−**45.08**	−41.57	−22.25	−**42.89**	−39.25
Glutamic acid	−27.96	58.04	25.92	−**69.74**	−33.62
Glutamine	**131.46**	**183.91**	**146.65**	−35.24	−20.57
Glycine	355.12	369.49	−51.58	−**67.22**	−66.18
Lysine	27.69	0.10	**131.07**	−**28.36**	−43.84
Methionine	−**48.33**	−**54.64**	5.56	−**58.85**	−**63.88**
Ornithine	47.41	49.10	15.80	−60.58	−**60.13**
Phenylalanine	−19.50	−21.98	193.75	−**62.24**	−**63.40**
Proline	−38.76	**380.99**	−58.27	−**86.46**	6.31
Pyroglutamic acid	7.94	−9.09	**55.14**	26.24	6.32
Serine	−21.05	221.43	−67.74	−**92.31**	−68.70
Threonine	**880.39**	2848.65	**2255.33**	−**78.26**	−34.62
Tyrosine	−25.86	145.79	**111.05**	−**68.75**	3.62
Valine	−10.26	−8.36	89.77	−**50.34**	−**49.29**
**Fatty Acids**					
Eicosanoic acid	28.42	−9.22	−3.42	**22.53**	−13.38
Heptadecanoic acid	**34.76**	−10.21	−11.78	26.59	−15.65
Hexadecanoic acid	**26.69**	−13.40	−9.58	**26.84**	−13.29
Octadecanoic acid	**20.06**	−11.20	−6.53	**19.79**	−11.39
Tetradecanoic acid	7.02	−1.86	−9.56	−1.62	−9.77
**Lipids**					
Docosenoic acid methyl ester, 13-(Z)-	−83.02	−28.84	36.45	−69.92	26.04
Squalene, all-trans-	−36.30	7.05	NA	−0.03	68.01
**N- Compounds**					
Agmatine	12.38	**53.02**	66.80	19.88	**63.22**
Arginine	−1.41	−48.47	164.72	−44.79	−**71.14**
Ethanolamine	−2.70	17.03	−0.90	−12.56	5.18
Hydroxyurea	NA	NA	−44.61	NA	NA
Putrescine	33.58	**82.10**	63.06	9.04	**48.64**
Pyridine, 2,3-dihydroxy-	2.06	18.85	8.25	0.61	17.16
Pyridine, 2-hydroxy-	−4.42	16.54	5.35	−8.41	11.68
Pyridine, 3-hydroxy-	−26.86	11.94	1.72	−23.01	17.83
Swainsonine	9.64	−5.22	−6.63	8.27	−6.40
**Phenylpropanoids**					
Sinapic acid, cis-	−23.42	144.74	−10.87	−60.12	27.46
Sinapic acid, trans-	−29.00	**213.57**	−10.44	−**70.07**	32.18
**Phosphates**					
Fructose-6-phosphate	−15.65	37.23	14.21	23.27	100.55
Glucose-6-phosphate	0.35	−22.32	**125.93**	**75.44**	35.82
Glyceric acid-3-phosphate	−12.77	−10.20	**110.76**	39.30	43.41
Glycerol-3-phosphate	214.50	NA	−17.41	NA	−46.80
Glycerophosphoglycerol	11.52	**216.07**	21.56	−33.95	87.21
myo-Inositol-1-phosphate	−28.61	80.52	−37.42	−23.58	93.25
Phosphoric acid	−**54.25**	−**47.71**	15.09	7.35	22.69
Phosphoric acid monomethyl ester	−14.80	16.87	45.51	−4.52	30.96
**Polyhydroxy Acids**					
Arabinonic acid	15.41	**86.67**	32.71	−**66.45**	−**45.73**
Erythronic acid	−34.46	137.32	−28.55	−**53.86**	67.05
Galactonic acid	−17.49	62.35	52.85	−**44.94**	8.35
Gluconic acid	−19.79	**181.13**	65.92	−**53.00**	**64.72**
Glyceric acid	−11.32	118.13	−12.66	−**68.31**	−22.05
Saccharic acid	−20.58	NA	73.52	NA	116.41
Threonic acid	**45.32**	**390.89**	70.20	−**61.72**	29.33
**Polyols**					
Erythritol	−**31.81**	−**29.23**	−31.72	−9.69	−6.27
Glycerol	−27.56	21.20	−**17.72**	−36.54	6.17
Inositol, myo-	−**37.47**	80.89	2.38	−**28.79**	106.00
**Sugars**					
4-Hydroxyphenyl-beta-glucopyranoside	−38.44	54.04	NA	−23.28	91.98
Fructose	−18.88	−**63.94**	29.47	43.30	−36.29
Glucose	−23.85	−**62.87**	−3.98	30.77	−36.23
Kestose, 1-	−85.31	−9.59	18.32	−79.87	23.93
Maltose	20.10	334.49	−12.14	−17.23	199.43
Raffinose	NA	NA	−47.82	NA	NA
Salicin	−20.07	NA	NA	NA	NA
Salicylaldehyde-beta-D-glucopyranoside	−5.57	40.65	8.30	−**44.97**	−18.04
Salicylic acid-glucopyranoside	40.22	NA	NA	NA	−36.75
Sucrose	2.39	−**33.85**	**73.48**	**131.46**	49.53
Trehalose, alpha,alpha'-, D-	−2.35	69.42	−4.51	−1.03	71.73

Plants were grown under 2 mM threonine or normal growth conditions for 8 days. Polar metabolites were then extracted and analyzed by GC-MS. Qualitative and quantitative Identification performed by Tagfinder. Post standardizing and normalizing, statistical analysis were performed using MeV. Bold values represent those that are statistically significant (P<0.05, students t-test). NA represents unavailability of data in those particular samples. Fold Change (FC) of the metabolites are presented here. Those that are highlighted bold are statistically significant, p<0.05 (student's t-test).

Thirteen compounds (ten identifiable metabolites and three mass spectral tags) that varied between *glx2-1* plants grown in the presence or absence of threonine were identified through comparison of the metabolic profiles (Table 1 and Table S1). All of the identified metabolites were elevated in *glx2-1* plants subjected to threonine stress. Of the ten metabolites that could be identified, six belonged to the amino acid class of compounds. They are threonine, lysine, valine, pyroglutamic acid, asparagine and glutamine. The remaining four that increased under threonine stress were sucrose, glucose, glyceric acid-3-phosphate and glycerol.

Metabolic differences between *GLX2-1OE* plants grown under control and stress conditions were also compared. Under threonine stress, fifteen metabolites and eight mass spectral tags were observed to accumulate *in GLX2-1OE* plants. The primary classes of these metabolites were amino acids, organic acids, polyhydroxy acids and polyamines (Table 1 and Table S1). Glycine, glutamine, proline and threonine levels increased between 3 and 29 fold, with threonine showing the largest increase. Several TCA cycle intermediates, such as malic acid, fumaric acid and 2-oxo-glutaric, and polyhydroxy acids, including arabinonic acid, erythronic acid, gluconic acid and threonic acid, also increased in GLX2-1OE plants under threonine stress. Increases in agmatine and putrescine levels, which are known markers for the abiotic stress response [52], were also observed. Several nitrogenous compounds were also elevated in GLX2-1OE plants under threonine stress, while several primary sugar molecules were reduced. Glucose, fructose, sucrose were reduced by more than half in the GLX2-1OE plants under threonine stress. Methionine was the only amino acid that decreased under the stress conditions.

A comparison between metabolite profiles of *GLX2-1OE* and wild type plants grown under threonine stress showed that wild type plants accumulate several amino acids, including aspartic acid, asparagine, ornithine, arginine, methionine, and phenylalanine (Table 1). Polyamines and polyhydroxy acids also accumulate differently between wild type and *GLX 2-1OE* plants under threonine stress. While the levels of gluconic acid decrease, there is an increase in arabinonic acid, agmatine and putrescine in *GLX 2-1OE* plants compared to wild type plants under threonine stress. Variations in the levels of sugars and organic acids were not observed between *GLX 2-1OE* and wild type plants grown under threonine stress.

The levels of a relatively large number of metabolites were reduced in *GLX2-1OE* plants relative to wild-type plants under control conditions. Twenty-eight identifiable metabolites and nine mass spectral tags were reduced in *GLX2-1OE* plants, while only ten (five metabolites and five mass spectral tags) were increased (Table 1 and Table S1). Amino acids were the largest class of compounds reduced in *GLX2-1OE* plants relative to wild type under normal growth conditions. Glycine, valine, proline, asparagine, glutamine, serine, threonine, methionine, phenylalanine, lysine and ornithine levels were all higher in wild type plants compared to *GLX2-1OE* plants. Furthermore, several TCA cycle intermediates, including malic acid, citric acid, fumaric acid, glyceric acid, 2-oxo-glutaric acid were present in higher quantities in wild type plants relative to *GLX2-1OE* plants. Polyhydroxy acids, including arabinonic acid, malonic acid, gluconic acid, and erythronic acid were also reduced in *GLX 2-1OE* plants. In contrast, *GLX 2-1OE* plants contained higher levels of sucrose and glucose-6-phosphate, along with fatty acids like eicosanoic acid, hexadecanoic, and octadecanoic acid and several mass spectral tags compared to wild type plants under control conditions.

To understand how wild type plants alter their metabolism when exposed to threonine stress, the profiles of wild type plants grown under control conditions and threonine stress were compared. Fourteen identifiable metabolites and six mass spectral tags differed between wild type plants grown under control and threonine stress conditions. Of the fourteen metabolites, threonine, 2-oxo-glutaric acid, glutamine, hexa-, hepta-, and octa-deacanoic acid levels were higher under threonine stress, while the levels of 3-hydroxy-pyridine, phosphoric acid, 4-amino-butanoic acid, malonic acid, erythritol, myo-inositol, methionine, and fumaric acid were reduced (Table 1). Unlike *glx 2-1* and GLX2-1 OE plants, there is no clear variation in the levels of primary sugars or amino acids in wild type plants exposed to threonine stress.

## Discussion

In this study, the functional role of *Arabidopsis* GLX2-1 was investigated by determining the effect of its inactivation and over-expression on both plant growth and metabolite levels. Results presented here show that the protein does not play a critical role under normal growth conditions, but does serve an important role in stress response and/or recovery. Changes in GLX2-1 expression also affect the levels of several amino acids, polyamines and organic acids by a hitherto unknown mechanism.

Expression analyses of *GLX2-1, GLX2-2*, and *GLX2-5* using Genevestigator with publically-available microarray datasets show that Arabidopsis *GLX2* genes are up-regulated to varying degrees during multiple abiotic stresses. These include anoxia, hypoxia, drought, light, osmotic and salt stress (Figures S1 and S2). Our observations that *glx2-1* plants are more sensitive to salt stress and anoxia than wild type plants and that GLX2-1-OE plants are more resistant than wild type to anoxic stress (Figure 4), suggests that, GLX2-1 plays an important role in stress response and/or recovery in plants, similar to the situation for GLX2 enzymes. Furthermore, the observation that *glx2-1* plants are most sensitive during the recovery stage after anoxic stress suggests that it may play a role in detoxification of toxic products generated during anoxic stress.

In addition to being sensitive to salt and anoxic stress, *glx2-1* plants are also more sensitive than wild type plants to the presence of exogenous threonine, suggesting that a byproduct of threonine catabolism could be a target of GLX2-1. Unfortunately, the effect of threonine on plant growth appears to be complex and it is not known how excess threonine exerts an inhibitory effect. For example, exogenous threonine results in a clear reduction in shoot growth, but does not affect root growth (data not shown). A similar differential response between roots and shoots has been observed under hypoxic conditions [53]. Furthermore, we observed that, under control conditions, plants that over-express GLX2-1 also exhibit retarded shoot length similar to *glx2-1* plants (Figure 5). The mean shoot lengths in *glx2-1* and GLX2-1OE plants were 69% and 75% of those in wild type plants under control conditions, respectively. However, in the presence of exogenous threonine the mean root length of GLX2-1OE plants was identical to wild type, while in *glx2-1* plants it was only 33% of wild type. Therefore, while both inactivation and over-expression of GLX2-1 have a negative effect on shoot growth, inactivation of GLX2-1 causes increased sensitivity to excess threonine, while over-expression of GLX2-1 appears to have a modest protective effect.

To better understand the effect of threonine on plant growth and the role of GLX2-1 during threonine stress, metabolic profiling experiments were conducted on wild type *glx2-1* and GLX2-1OE plants, both in the presence and absence of exogenous threonine. As expected, all plants grown in the presence of threonine showed increased levels of threonine (Table 1). The TCA cycle intermediates, malic acid, citric acid and 2-oxoglutaric acid, were also increased in both *glx2-1*and GLX2-1OE plants, with higher levels observed in GLX2-1OE plants. These metabolites may be increased in order to provide carbon backbones, specifically 2-oxoglutaric acid that can act as N-acceptors in amino transferase reactions. Degradation of excess threonine should require sequestration of N, a process that can be performed by the cannonical nitrogen assimilating GOGAT system that detoxifies free ammonium through glutamic acid/glutamine and requires 2-oxoglutaric acid [54]. Consistent with either or both of these assumptions, glutamine, glutamate, and putrescine, a product of these amino acids, were increased under threonine stress in both *glx2-1* and GLX2-1OE plants, although at higher levels in GLX2-1OE plants. An alternative metabolic product of glutamate is the signalling and GABA-shunt constituent 4-aminobutyric acid. This metabolite was reduced in response to excess L-threonine in contrast to the metabolic neighbours of glutamine/glutamate.

During threonine stress *glx2-1* plants accumulate higher levels of the amino acids glutamine, lysine, valine, arginine, and threonine relative to both GLX2-1OE and wild type plants. Since lysine and arginine are known to sequester a high proportion of nitrogen relative to carbon they may be used by *glx2-1* plants to store surplus nitrogen, which may not be required in GLX2-1OE and wild type plants. Likewise, asparagine, aspartate and methionine are reduced in GLX2-1OE and wild type plants subjected to threonine stress, whereas they are elevated in *glx2-1* plants. This suggests that wild type and GLX2-1OE plants may be able to down-regulate the biosynthesis of the aspartate family of amino acids while *glx2-1* plants appear not to have this option.

Levels of the polyhydroxy acids, including threonic, arabinonic, galactonic and gluconic acid were increased in GLX2-1OE plants under threonine stress relative to wild type plants. A higher proportion of these acids should provide reducing equivalents as they are ulitmately generated from sugar-aldehydes. A system that can provide more carbon acceptor molecules, such as 2-oxoglutarate, should be able to better deal with the requirement for assimilating excess N. The decrease in sugars and increase in TCA cycle intermediates suggests that GLX2-1OE plants may process higher levels of soluble carbohydrate resources during threonine stress. This potential to shift carbon resources towards the TCA cycle may explain the increase in 2-oxoglutarate, malate and fumarate.

Proline, serine and glycerate are also elevated in GLX 2-1OE plants subjected to threonine stress (Table 1). Proline is a marker of environmental stress, whereas serine and glycerate are part of the photorespiratory pathway. Plants increase their tolerance to abiotic stress by accumulation of benign compounds like mannitol, proline, glycine and betaine [55], [56]. These observations suggest that GLX2-1OE plants may exhibit increased stress responses. GLX 2-1OE plants also accumulate basic phenylpropanoids, such as the cis- and trans-sinapic acids, as well as glycerol, glycerol-3P, erythronic acid, fumaric acid, maltose and a,a-trehalose. Increased levels of these compounds, especially maltose, may hint at an enhanced potential for starch mobilization when exposed to L-threonine stress. Taken together, the metabolic profiling data appear to indicate the differential potential of *glx2-1* and *GLX2-1OE* plants to assimilate N, which is derived from excess L- threonine. G*lx2-1* plants might be limited in their ability to adjust to excess L-threonine and may accumulate toxic levels of nitrogen. GLX 2-1OE plants have higher levels of soluble sugars under normal conditions, which appear to be used for the synthesis of the N-acceptor 2-oxoglutarate.

However, our metabolite screening did not identify a substrate for GLX-2-1 and the enzymatic function of GLX2-1 remains undefined. It is possible that a moderate substrate accumulation is sufficient to generate toxic levels, or that a moderate product depletion may interfere with the maintenance of an non-stressed metabolic state. It is also possible that the reduction of a GLX2-1 mediated flux, which cannot be diagnosed by the current pool size analyses, may trigger the enhanced stress sensitivity of *glx2-1* plants. Finally, and perhaps most likely, the GC-MS profiling may not cover the *in vivo* substrates and products of GLX2-1. Comparative time course analyses, especially of the anoxic response and of the recovery from anoxic stress using a multilevel metabolomic and transcriptomic approach should help reveal the primary role of GLX-2-1 and the apparently secondary responses in primary metabolism revealed by this study.

## Supporting Information

Figure S1 RT-PCR on total RNA isolated from the wild type, *glyoxalase2-1* and over- expression plants (Wt: wild type, KO: knockout, OE: over-expression line 5).
(PDF)

Figure S2 Response from Genevestigator for query on expression pattern of Glyoxalase 2-1 (AT2g43430) shows that enhanced expression is observed for various external stimuli studied. (Image truncated for clarity).
(PDF)

Figure S3 Genevestigator analysis of *GLX 2-1*, *GLX 2-2* and *GLX 2-5*. Differential expression to various stimuli in decreasing order is shown. (Image Truncated for clarity).
(PDF)

Figure S4 Co-expression network analyses as carried out by ATTED II. Genes that exhibit a correlation in expression similar to *A.thaliana GLX 2-1* (timing of expression, site of expression, level of expression) are depicted in network form (a). The thicker the lines, the stronger the mutual ranking. The list of top ten GLX2-1 co-expressed genes and their annotated roles are shown (b). Detailed look into the top 300 gene list reveals that many of these co expressed genes play role in stress response.
(PDF)

Figure S5 Motif alignment showing some of the stress responsive cis-regulatory elements found upstream of *GLX 2-1*. The motif alignment was obtained by screening for common motifs using the tool AtCOECiS [54] (http://bioinformatics.psb.ugent.be/ATCOECIS/). Stress motifs are highlighted with specific binding sites noted below. The *GLX 2-1* ATG coding region is presented in CAPS and the initiation codon in red font.
(PDF)

Figure S6 Screen shot of Mapman, a gene to pathway mapping tool. Small squares represent specific genes. Their placement in the specific pathway is based on several factors as described elsewhere [34]. A search for *GLX 2-1* in the default metabolism data set reveals that the gene is placed under amino acid metabolism, specifically, metabolism/catabolism of aspartate family of amino acids. Image truncated for clarity.
(PDF)

Table S1 Variations in signature mass spectral tags between plants grown under different conditions and differential for GLX2-1 expression.
(DOCX)
