# Supplementary figures and images for: Arabidopsis thaliana Glyoxalase 2-1 Is Required during Abiotic Stress but Is Not Essential under Normal Plant Growth
Source: PLoS One. 2014 Apr 23;9(4):e95971. doi: 10.1371/journal.pone.0095971 (PMC3997514; doi:10.1371/journal.pone.0095971)

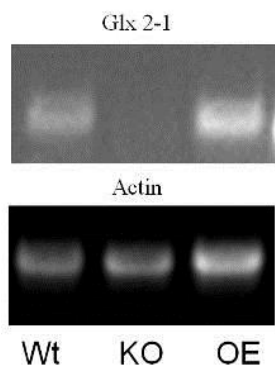

**Figure S1**

Supplement: Figure S1 — RT-PCR on total RNA isolated from the wild type, glyoxalase2-1 and over- expression plants (Wt: wild type, KO: knockout, OE: over-expression line 5). (PDF) [file pone.0095971.s001.pdf]

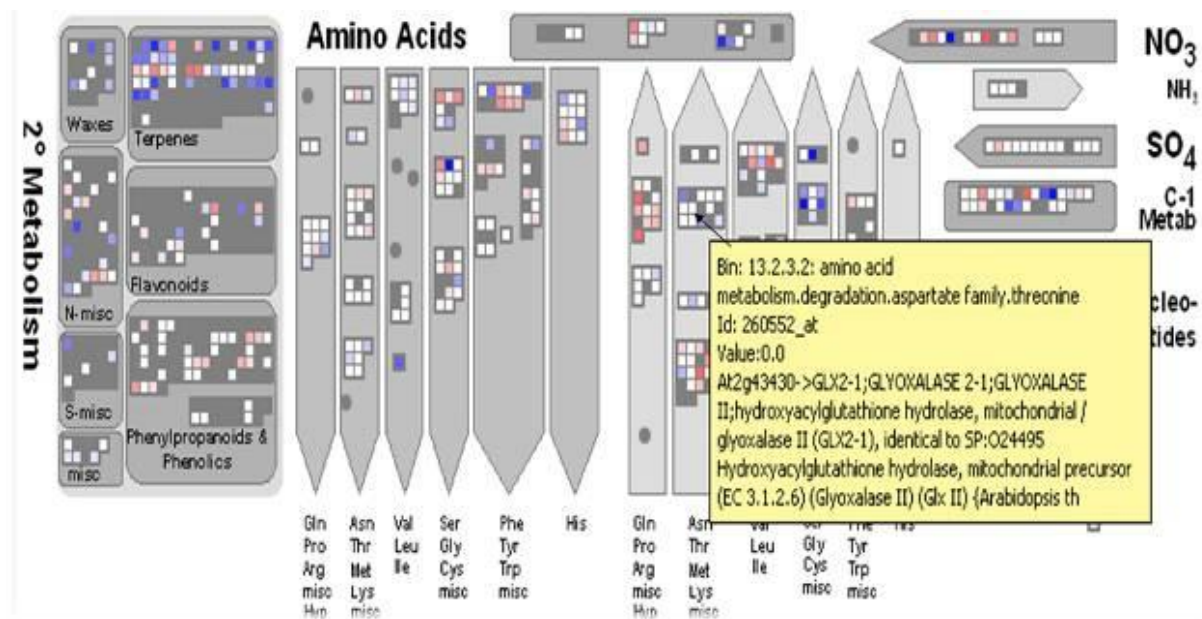

Figure S6

Supplement: Figure S6 — Screen shot of Mapman, a gene to pathway mapping tool. Small squares represent specific genes. Their placement in the specific pathway is based on several factors as described elsewhere [34]. A search for GLX 2-1 in the default metabolism data set reveals that the gene is placed under amino acid metabolism, specifically, metabolism/catabolism of aspartate family of amino acids. Image truncated for clarity. (PDF) [file pone.0095971.s006.pdf]
