# Supplementary material for: Arabidopsis thaliana Glyoxalase 2-1 Is Required during Abiotic Stress but Is Not Essential under Normal Plant Growth
Source: PLoS One. 2014 Apr 23;9(4):e95971. doi: 10.1371/journal.pone.0095971 (PMC3997514; doi:10.1371/journal.pone.0095971)
Supplement: Figure S3 — Genevestigator analysis of GLX 2-1, GLX 2-2 and GLX 2-5. Differential expression to various stimuli in decreasing order is shown. (Image Truncated for clarity). (PDF) [file pone.0095971.s003.pdf]

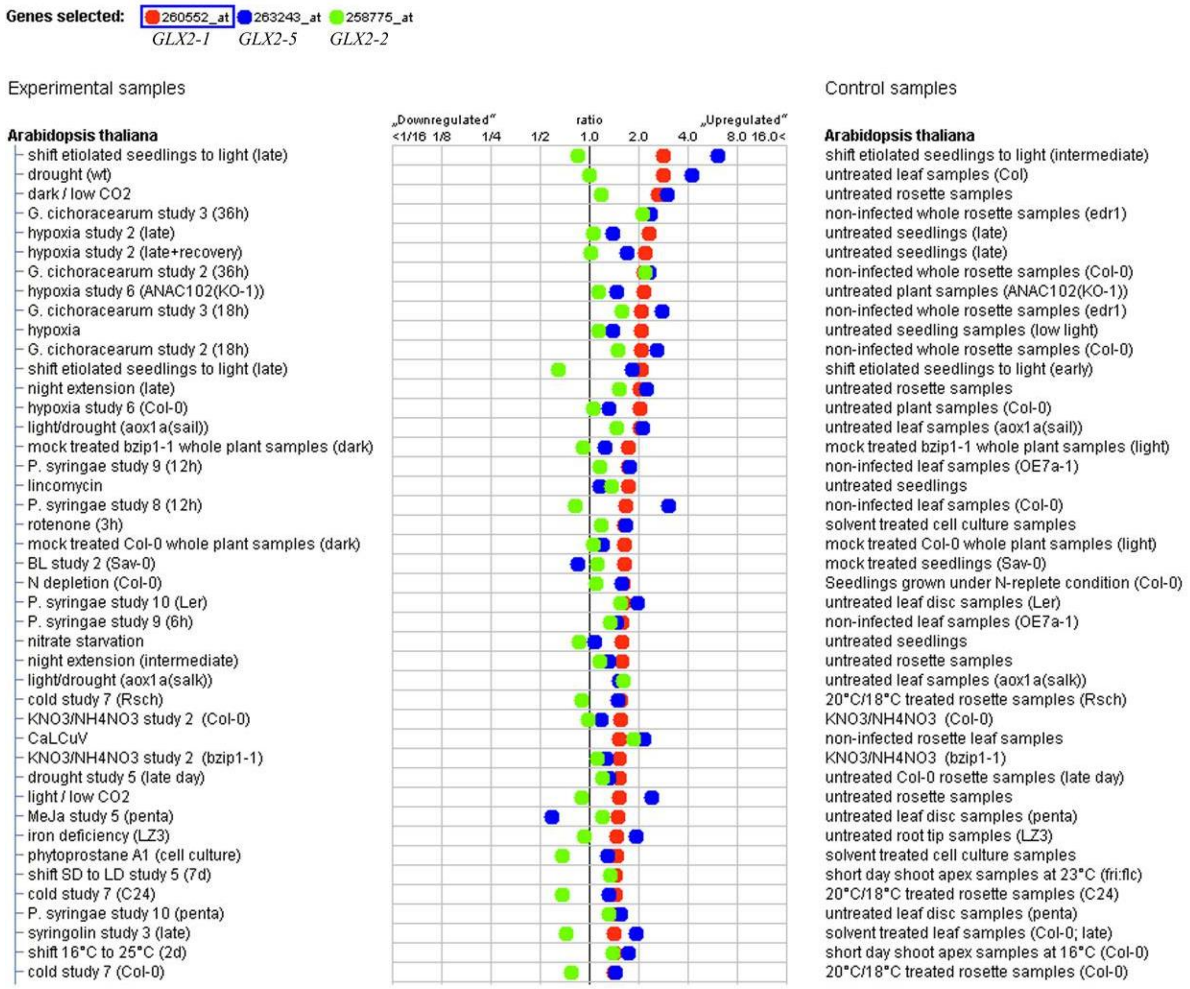
Genes selected:
260552_at
263243_at
258775_at
GLX2-1
GLX2-5
GLX2-2
Experimental samples
Control samples
„Downregulated"
ratio
„Upregulated"
<1/16 1/8 1/4 1/2 1.0 2.0 4.0 8.0 16.0<
Arabidopsis thaliana
shift etiolated seedlings to light (late)
drought (wt)
dark / low CO2
G. cichoracearum study 3 (36h)
hypoxia study 2 (late)
hypoxia study 2 (late+recovery)
G. cichoracearum study 2 (36h)
hypoxia study 6 (ANAC102(KO-1))
G. cichoracearum study 3 (18h)
hypoxia
G. cichoracearum study 2 (18h)
shift etiolated seedlings to light (late)
night extension (late)
hypoxia study 6 (Col-0)
light/drought (aox1a(sail))
mock treated bzip1-1 whole plant samples (dark)
P. syringae study 9 (12h)
lincomycin
P. syringae study 8 (12h)
rotenone (3h)
mock treated Col-0 whole plant samples (dark)
BL study 2 (Sav-0)
N depletion (Col-0)
P. syringae study 10 (Ler)
P. syringae study 9 (6h)
nitrate starvation
night extension (intermediate)
light/drought (aox1a(salk))
cold study 7 (Rsch)
KNO3/NH4NO3 study 2 (Col-0)
CaLCuV
KNO3/NH4NO3 study 2 (bzip1-1)
drought study 5 (late day)
light / low CO2
MeJa study 5 (penta)
iron deficiency (LZ3)
phytoprostane A1 (cell culture)
shift SD to LD study 5 (7d)
cold study 7 (C24)
P. syringae study 10 (penta)
syringolin study 3 (late)
shift 16°C to 25°C (2d)
cold study 7 (Col-0)
Arabidopsis thaliana
shift etiolated seedlings to light (intermediate)
untreated leaf samples (Col)
untreated rosette samples
non-infected whole rosette samples (edr1)
untreated seedlings (late)
untreated seedlings (late)
non-infected whole rosette samples (Col-0)
untreated plant samples (ANAC102(KO-1))
non-infected whole rosette samples (edr1)
untreated seedling samples (low light)
non-infected whole rosette samples (Col-0)
shift etiolated seedlings to light (early)
untreated rosette samples
untreated plant samples (Col-0)
untreated leaf samples (aox1a(sail))
mock treated bzip1-1 whole plant samples (light)
non-infected leaf samples (OE7a-1)
untreated seedlings
non-infected leaf samples (Col-0)
solvent treated cell culture samples
mock treated Col-0 whole plant samples (light)
mock treated seedlings (Sav-0)
Seedlings grown under N-replete condition (Col-0)
untreated leaf disc samples (Ler)
non-infected leaf samples (OE7a-1)
untreated seedlings
untreated rosette samples
untreated leaf samples (aox1a(salk))
20°C/18°C treated rosette samples (Rsch)
KNO3/NH4NO3 (Col-0)
non-infected rosette leaf samples
KNO3/NH4NO3 (bzip1-1)
untreated Col-0 rosette samples (late day)
untreated rosette samples
untreated leaf disc samples (penta)
untreated root tip samples (LZ3)
solvent treated cell culture samples
short day shoot apex samples at 23°C (fri:flc)
20°C/18°C treated rosette samples (C24)
untreated leaf disc samples (penta)
solvent treated leaf samples (Col-0; late)
short day shoot apex samples at 16°C (Col-0)
20°C/18°C treated rosette samples (Col-0)

**Figure S3**
