# Supplementary material for: Arabidopsis thaliana Glyoxalase 2-1 Is Required during Abiotic Stress but Is Not Essential under Normal Plant Growth
Source: PLoS One. 2014 Apr 23;9(4):e95971. doi: 10.1371/journal.pone.0095971 (PMC3997514; doi:10.1371/journal.pone.0095971)
Supplement: Figure S4 — Co-expression network analyses as carried out by ATTED II. Genes that exhibit a correlation in expression similar to A.thaliana GLX 2-1 (timing of expression, site of expression, level of expression) are depicted in network form (a). The thicker the lines, the stronger the mutual ranking. The list of top ten GLX2-1 co-expressed genes and their annotated roles are shown (b). Detailed look into the top 300 gene list reveals that many of these co expressed genes play role in stress response. (PDF) [file pone.0095971.s004.pdf]

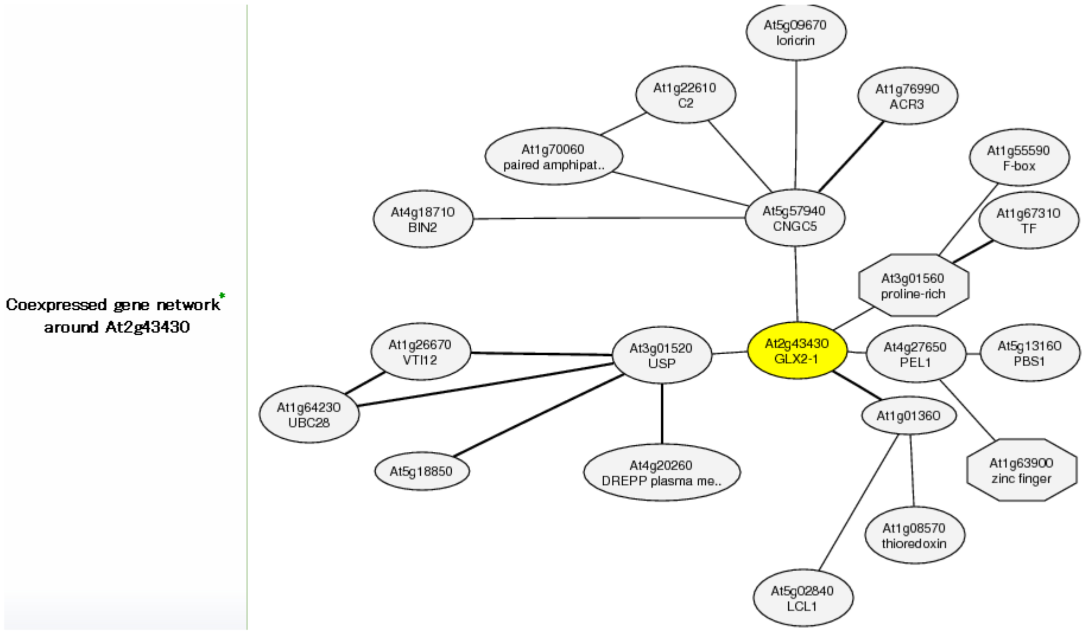

a)

	Locus*	Alias* (short description)	Function*	MR* (all) [sort]
0	At2g43430	GLX2-1	GLX2-1 (GLYOXALASE 2-1); hydroxyacylglutathione hydrolase	**0.0**
1	At1g01360	RCAR1	unknown protein	**3.0**
2	At5g57940	CNGC5	ATCNGC5 (CYCLIC NUCLEOTIDE GATED CHANNEL 5); calmodulin binding / cyclic nucleotide binding / potassium channel	**6.3**
3	At3g01520	USP	universal stress protein (USP) family protein	**9.2**
4	At5g14680	USP	universal stress protein (USP) family protein	**9.8**
5	At1g25420		unknown protein	**10.2**
6	At2g35050	kinase	protein kinase family protein	**11.6**
7	At1g70900		unknown protein	**11.9**
8	At4g02440	EID1	EID1 (EMPFINDLICHER IM DUNKELROTEN LICHT 1); ubiquitin-protein ligase	**13.6**
9	At3g01560	proline-rich	proline-rich family protein	**13.9**
10	At4g35780	kinase	protein kinase family protein	**15.8**

b)

Figure S4
