# Supplementary material for: Arabidopsis thaliana Glyoxalase 2-1 Is Required during Abiotic Stress but Is Not Essential under Normal Plant Growth
Source: PLoS One. 2014 Apr 23;9(4):e95971. doi: 10.1371/journal.pone.0095971 (PMC3997514; doi:10.1371/journal.pone.0095971)
Supplement: Figure S5 — Motif alignment showing some of the stress responsive cis-regulatory elements found upstream of GLX 2-1. The motif alignment was obtained by screening for common motifs using the tool AtCOECiS [54] (http://bioinformatics.psb.ugent.be/ATCOECIS/). Stress motifs are highlighted with specific binding sites noted below. The GLX 2-1 ATG coding region is presented in CAPS and the initiation codon in red font. (PDF) [file pone.0095971.s005.pdf]

5’…ttgtcaataagatagagatatgcacaattcttaccgaactattttggtattgaactctatatattttcttcaactttcaattgagataaagaaaaaag
ataatgattataaaagaatctcaacaaactaaatatacacatcccagaatctagatattatcaactaatgttaaaagtttcaatttatgataactgatat
aattaatgcttttttttttcttacttgataagataaattttatatattctataaaaacccaaatgatcaaggcaagcgtttaatctaaattgaccttaacca
aatgagcaaatattgaccaaaggaaatcttttctatttttttgcgtttatcctaaaggaaatgtttgtttcatggcttcaaaagggaatataatttgagca
aaacaagtaaaaaagtaacaaattaccgttacagcaaaaacaactttaataaataataacaga gcatatggtaacaactttattacccaattcaaa

(GARE) (ERELEE4ERE)

taaagaaaataataaaccgtggtcaaatatttgtatgaattatatgagatagcacggaacaatttattgatgggatttagtagttgagctaaataaata
aaaagcaaaaacaaataaaataaaacaaaatactaaagaaaaaaaaagagtaaaagctgtctc tttagctctgacgttgtttcctgtcgaagtcgc

(ARF)

agactcgtcatcacagctcaggcgagtaattctgagtttcttctatctacgcgcaatatctctcaaaggaggcctcttcaaaggtaccacaatcatatcc

(ANAERO3)

tctcgtttctatctgaagaatcagagagaagagatatatagaggATGCCTGTGATCTCCAAAGCTTCATCTACCACCACCAATTCAT
CGATTCCTTCGTGTTCTAGG……3’

Figure S5
