# Supplementary material for: Arabidopsis thaliana Glyoxalase 2-1 Is Required during Abiotic Stress but Is Not Essential under Normal Plant Growth
Source: PLoS One. 2014 Apr 23;9(4):e95971. doi: 10.1371/journal.pone.0095971 (PMC3997514; doi:10.1371/journal.pone.0095971)
Supplement: Table S1 — Variations in signature mass spectral tags between plants grown under different conditions and differential for GLX2-1 expression. (DOCX) [file pone.0095971.s007.docx]

**Table S1**

| **Mass Spectral Tags** | **Fold change (WTT/WTC) (n=6)** | **Fold change (OET/OEC)**  **(n=6)** | **Fold change (KOT/KOC) (n=6)** | **Fold change (OEC/WTC)**  **(n=6)** | **Fold change (OET/WTT) (n=6)** |
| --- | --- | --- | --- | --- | --- |
| A182009-101 | -46.32 | -86.69 | 22.36 | -52.08 | -88.12 |
| A180002-101 | -51.44 | -90.35 | 79.33 | 2.73 | -79.58 |
| A181004-101 | -47.18 | -89.90 | 87.30 | -22.15 | -85.11 |
| A139007-101 | 13.31 | 6.28 | 23.99 | 0.37 | -5.86 |
| A142003-101 | -30.49 | -41.75 | -61.08 | **246.38** | 190.23 |
| A143003-101 | -4.81 | -1.65 | -3.70 | -5.56 | -2.42 |
| A144006-101 | -4.70 | 3.59 | -3.58 | -8.00 | 0.01 |
| A145008-101 | **-86.79** | 44.58 | -4.10 | **-93.16** | -25.13 |
| A151008-101 | -16.91 | -73.18 | NA | 3.02 | -66.75 |
| A159003-101 | -8.14 | 426.65 | -51.08 | -74.09 | 48.55 |
| A165002-101 | 37.64 | 3.55 | 60.24 | -42.89 | -57.03 |
| A167004-101 | 134.52 | 378.76 | 90.63 | -56.58 | -11.35 |
| A168011-101 | 306.15 | 79.21 | 227.25 | -17.17 | -63.45 |
| A171003-101 | 23.56 | 208.86 | 23.28 | -47.28 | 31.79 |
| A174005-101 | **72.14** | 202.12 | 9.16 | -27.63 | 27.02 |
| A175008-101 | **55.00** | 176.22 | 11.44 | -24.72 | 34.16 |
| A176001-101 | -18.57 | 17.17 | 27.44 | **-40.21** | -13.97 |
| A178011-101 | 25.67 | 70.91 | 51.33 | **-57.29** | **-41.91** |
| A187005-101 | -13.81 | -4.96 | 37.80 | **-38.36** | -32.03 |
| A191007-101 | -21.67 | -66.99 | -21.54 | 9.35 | -53.92 |
| A203003-101 | -38.26 | 109.36 | -54.90 | -29.16 | 140.24 |
| A211001-101 | 7.19 | 190.61 | -10.78 | **-63.50** | -1.05 |
| A213001-101 | -9.94 | 1.44 | -7.91 | -11.90 | -0.77 |
| A214003-101 | NA | NA | NA | NA | -5.81 |
| A217004-101 | 0.49 | 156.91 | 21.36 | **-46.99** | 35.51 |
| A225007-101 | -17.21 | 28.85 | -2.09 | 5.81 | 64.68 |
| A237001-101 | 7.26 | 206.14 | 65.30 | **-66.24** | -3.65 |
| A241003-101 | 28.32 | 121.59 | 49.40 | 35.91 | 134.68 |
| A250001-101 | -1.07 | 87.02 | 21.52 | -31.77 | 28.99 |
| A254002-101 | -4.35 | 221.98 | 8.42 | **-56.37** | 46.87 |
| A254009-101 | NA | NA | NA | NA | NA |
| A300001-101 | **-29.01** | -20.78 | 8.93 | -19.10 | -9.71 |
| A302001-101 | 20.81 | 133.01 | 113.82 | 28.09 | 147.06 |
| A304001-101 | -13.43 | -67.43 | 20.81 | **162.22** | -1.36 |
| A306001-101 | -23.05 | -19.23 | 70.07 | 7.82 | 13.18 |
| A308003-101 | -16.70 | 45.27 | -1.27 | 70.37 | 197.11 |
| A311002-101 | -42.77 | 530.68 | -25.04 | -57.49 | 368.42 |
| A313001-101 | -3.52 | 76.27 | -19.55 | **83.76** | 235.73 |
| A317003-101 | -17.67 | 118.68 | -36.35 | 52.06 | 303.88 |
| A324001-101 | 12.27 | 199.18 | 5.22 | -24.76 | 100.51 |
| A221002-101 | 11.96 | -6.81 | -9.25 | **30.22** | 8.39 |
| A242003-101 | 14.18 | -10.75 | -5.17 | **29.24** | 1.02 |
| A296004-101 | -55.22 | 162.78 | 159.20 | **-72.15** | 63.44 |
| A318004-101 | 23.75 | 3.07 | -22.19 | 13.52 | -5.45 |
| A329006-101 | 1.50 | 242.83 | 5.44 | 3.03 | 247.99 |
| A112003-101 | **-80.21** | -12.96 | -19.68 | **-78.49** | -5.38 |
| A274014-101 | -41.16 | 186.29 | -18.18 | **-81.08** | -7.92 |

Plants were grown under 2mM threonine or normal growth conditions for 8 days. Polar metabolites were then extracted and analysed by GC-MS. Qualitative and quantitative Identification performed by Tagfinder. Post standardizing and normalizing, statistical analysis were performed using MeV. Bold values represent those that are statistically significant (P<0.05, students t-test).NA represents unavailability of data in those particular samples.
